# Genome comparison of different *Zymomonas mobilis* strains provides insights on conservation of the evolution

**DOI:** 10.1371/journal.pone.0195994

**Published:** 2018-04-25

**Authors:** Chen Chen, Linfeng Wu, Qinghua Cao, Huanhuan Shao, Xuedan Li, Yizheng Zhang, Haiyan Wang, Xuemei Tan

**Affiliations:** College of Life Sciences, Sichuan University, Key Laboratory for Bio-Resources and Eco-Environment of Ministry of Education, Sichuan Key Laboratory of Molecular Biology and Biotechnology, Chengdu, PR China; Hubei University, CHINA

## Abstract

*Zymomonas mobilis* has the special Entner-Doudoroff (ED) pathway and it has excellent industrial characteristics, including low cell mass formation, high-specific productivity,ethanol yield, notable ethanol tolerance and wide pH range, a relatively small genome size. In this study, the genome sequences of NRRL B-14023 and NRRL B-12526 were sequenced and compared with other strains to explore their evolutionary relationships and the genetic basis of *Z*. *mobilis*. The comparative genomic analyses revealed that the 8 strains share a conserved core chromosomal backbone. ZM4, NRRL B-12526, NRRL B-14023, NCIMB 11163 and NRRL B-1960 share 98% sequence identity across the whole genome sequences. Highly similar plasmids and CRISPR repeats were detected in these strains. A whole-genome phylogenetic tree of the 8 strains indicated that NRRL B-12526, NRRL B-14023 and ATCC 10988 had a close evolutionary relationship with the strain ZM4. Furthermore, strains ATCC29191 and ATCC29192 had distinctive CRISPR with a far distant relationship. The size of the pan-genome was 1945 genes, including 1428 core genes and 517 accessory genes. The genomes of *Z*. *mobilis* were highly conserved; particularly strains ZM4, NRRL B-12526, NRRL B-14023, NCIMB 11163 and NRRL B-1960 had a close genomic relationship. This comparative study of *Z*. *mobilis* presents a foundation for future functional analyses and applications.

## Background

*Zymomonas mobilis* is facultative anaerobic bacterium; it can grow in aerobic or anaerobic conditions [1, 2]. *Z*. *mobilis* has the special Entner-Doudoroff (ED) pathway and demonstrates high ethanol production using glucose, fructose or sucrose as substrates [3,4]. *Z*. *mobilis* has excellent industrial characteristics, including low cell mass formation, high-specific productivity and ethanol yield, notable ethanol tolerance and a wide pH range [5–7]. *Z*. *mobilis* has also been used for a variety of other biotechnological purposes, such as the production of levan [8, 9].

Recently, different genetic techniques were used to improve the industrial biotechnology capacity of *Z*. *mobilis* [10, 11]. Genome sequencing analysis of *Z*. *mobilis* provides further opportunities for strain developments and fundamental research [12]. The first genome sequence of the *Z*. *mobilis* ZM4 was published in 2005[10]. This strain is regarded as a model organism in *Z*. *mobilis* research and industrial applications [12, 13]. Thus far, the complete genome sequences of 9 *Z*. *mobilis* strains have been reported, including two sequences for *Z*. *mobilis* NRRL B-14023 (CP4) [14–19]. In fact, the genomes of *Z*. *mobilis* strains NRRL B-12526 and NRRL B-14023 were sequenced in our lab. Draft genome sequences of two *Z*. *mobilis* strains (ATCC 31822 and ATCC 31823) have also been reported [20,21]. Thus, in this article, we chose 8 *Z*. *mobilis* strains with complete genome sequences to analyze and compare (Table 1).

**Table 1:** Sequenced *Z*. *mobilis* strains and available genomes used in this study.

Strain	NCBI accession NO	Size (Mb)	GC%	Gene	Protein	plasmid number	origin	reference
ZM4	NC_006526.2	2.06	46.3	1819	1746	5	Recife, Brazil	[12]
NRRL B-14023	NZ_CP003715.1	2.01	46.2	1776	1708	5	China	-
NRRLB- 12526	NZ_CP003709.1	2.01	46.2	1776	1708	4	China	-
ATCC 29192	NC_015709.1	1.99	44.1	1761	1673	2	Bristol, United Kingdom	[20]
NCIMB 11163	NC_013355.1	2.12	46.8	1879	1797	3	United Kingdom	[14]
ATCC 10988	NC_017262.1	2.02	46.2	1815	1731	6	Mexican	[17]
ATCC 29191	NC_018145.1	1.96	46.2	1787	1684	3	Zairian Elaeis	[15]
NRRL B-1960	CP021053	2.05	46.1	1804	1713	2	United Kingdom	[16]

Currently, three subspecies (subsp.) of *Z*. *mobilis* have been found, including “*Z*. *mobilis* subsp. *mobilis”*, “*Z*. *mobilis* subsp. *pomaceae”* and “*Z*. *mobilis* subsp. *francensis”*. [21–23]. ZM4, ATCC 10988, ATCC29191, NRRL B-14023, NRRL B-12526, NCIMB 11163 and NRRL B-1960, belonge to *Z*. *mobilis* subsp *mobilis* [21–23]. ATCC 29192 is the type strain of *Z*. *mobilis* subsp. *pomaceae* [20].

Moreover, different srtains of subspecies have different characteristics. For example, NRRL B-14023 is the most aerotolerant, quickly growing, and ethanol-yielding *Z*. *mobilis* strain [18]. ATCC 29191 is superior to other *Z*. *mobilis* strains in levan production [15]. ATCC 29192 exhibits distinct traits compared to other strains, including low oxygen tolerance, increased nutritional requirements, inability to utilize sucrose, and low DNA hybridization relatedness [20]. Differences of these strains in physiological and fermentation ability may be related to its genome. Although the complete genome sequences of these strains had been published, the comparative genome analysis has not been reported. Comparative genomics analysis of the genomes of different strains simultaneously could identify similarities and differences among them, which could be helpful for a better understanding of the genetic relationships among strains. The results will provide insights on the evolutionary relationships of different *Z*. *mobilis* strains and provide guidance for strain engineering to improve ethanol production.

In this study, the genomes of *Z*. *mobilis* strains NRRL B-12526 and CP4 = NRRL B-14023 were sequenced in our lab, and comparative genomics was analyzed among 8 *Z*. *mobilis* stains. Our aim was to perform comparative genomics analyses on these sequence data to find evolutionary relationships in *Z*. *mobilis*.

## Materials and methods

### Strains, genome sequencing, assembly, annotation and whole-genome comparison

The *Z*. *mobilis* strains of NRRL B-14023 and NRRL B-12526 were purchased from China Center of Industrial Culture Collection (CICC). Genomic DNA was extracted from strains using the Gentra Pure Gene Blood kit (Qiagen) according to the manufacturer's instructions. DNA was sent to the Beijing Genomics Institute (BGI)-Shenzhen, Shenzhen, China (http://www.genomics.cn) for whole genome sequencing. In addition, we also downloaded the genomic data for *Z*. *mobilis* gathered across the world from the EMBL-EBI database (ftp.sra.ebi.ac.uk/vol1/fastq/ERR173/) (Table 1).

After Sequencing completed, original file of fastq format was download from BGI company ftp server and assessed reads quality with FastQC (loaded in http://www.bioinformatics.bbsrc.ac.uk/projects/download.html). To obtain the best assembled results, we employed two assembly software programs to assemble the genome of *Z*.*mobilis*, including Edena V3 [24] and Velvet [25].These scaffolds were ordered relative to the genome of the *Z*. *mobilis* strain ZM4, using a combination of the Contiguator [26] and ACT 10.2.0 [27]. The Illumina reads were remapped to the scaffolds using Bowtie 0.12.7[28] and visualized by Tablet 1.12.02.06[29]. Annotation was primarily done by Prokka[30], which uses Prodigal 2.6 [31] to predict gene sequence similarity analysis. GO annotations were assigned by Blast2GO[32]. Mauve was applied for whole chromosome genome comparison and plasmid genome comparison [33]. A circular genomic map for the genomes of 8 *Z*. *mobilis* strains genomes were compared using the BLAST Ring Image Generator (BRIG) [34].

### Phylogeny tree construction

To better understand the evolutionary relationships and genomic variations at the gene level, the phylogenetic relationship of the *Z*. *mobilis* strains were constructed based on the complete genome sequences using MEGA6 software [35].

### Comparative and pan-genome analysis

A total of 8 *Z*. *mobilis* genome sequences and protein sequences were downloaded from NCBI (Table 1). Pan-genome analysis was performed on a larger dataset of these 8 *Z*. *mobile* genomes using the Gene Family method in the pan-genome analysis pipeline. All proteins were filtered with the criteria of 50% coverage and 50% identity, and ortholog clusters were generated using MCL software.

Core-genome and pan-genome calculations were performed as previously described by Liu [36]. Orthologous protein sequences among the eight *Z*. *mobilis* genomes were defined by OrthoMCL version 2.0 [37]. Briefly, estimations of core genes, new genes, and pan-genome size were performed using all-against-all BLASTp search within and between all genome pairs and all-versus-all WU-TBLASTN searches. Homologous clusters from OrthoMCL were compiled to identify shared and unique genes [36]. The core genes, new genes, and pan-genome size were calculated for each combination and then extrapolated using several functions to find a best fit from the mean number at each sampling point [36, 38].

### CRISPR-Cas system

The genome sequences of all eight *Z*. *mobilis* strains were analyzed for CRISPR repeats using CRISPRdb [39]. CRISPRs Finder (http://crispr.u-psud.fr/) was used to identify clustered regularly interspaced short palindromic repeats (CRISPRs) [40].

### Accession numbers

The genome sequences of *Z*. *mobilis* subsp. *mobilis* strains CP4 = NRRL B-14023 and NRRL B-12526 were deposited into the GenBank under the accession numbers of NZ_CP003715.1 (chromosome) and NC_CP003711.1-NC_CP003715.1 (plasmids); NZ_CP003709.1 (chromosome) and NC_CP003716.1-NC_CP003719.1(plasmids), respectively.

## Results and discussion

### General genomic features and plasmids of *Z*. *mobilis* NRRL B-12526 and NRRL B-14023

NRRL B-12526 was composed of a circular chromosome of 1,998,163bp and 5 circular plasmids, pZM1252601 to pZM1252605, which were 33,915bp, 30,952bp, 37,058bp, 32,400 bp and 32,801bp, respectively. The entire genome contained 1,708 protein-coding genes, 51 tRNA genes, and 9 rRNA gene clusters. NRRL B-14023 contained a circular chromosome of 2,012,538 bp and 4 plasmids, pZM1402301 to pZM1402304, which were 33,915bp, 30,952bp, 37,058bp and 32,801bp, respectively. We identified that NRRL B-12526 and NRRL B-14023 genomes shared an average 99.5% identity at the nucleotide level. The G + C content for strains NRRL B-12526 and NRRL B-14023 were 50.8% and 50.7%, respectively. The characteristics of genomes and plasmids were shown in Table 1 and Table 2.

**Table 2:** Plasmid characters of *Z*. *mobilis* strains used in this study.

Strains	Plasmid name	RefSeq	Size (Kb)	GC(%)	Protein	Gene	Pseudo-gene
ZM4 = ATCC 31821	pZZM401	NC_013356.1	37.07	42.4	53	51	2
	pZZM402	NC_013357.1	33.92	42.3	33	30	3
	pZZM403	NC_013358.1	32.8	43.3	25	21	4
	pZZM404	NC_017180.1	32.4	43.7	31	28	3
	pZZM405	NC_017183.1	30.95	43.7	27	26	1
NRRL B-12526	pZM1252601	NC_CP003711.1	33.92	42.3	29	28	1
	pZM1252602	NC_CP003712.1	30.95	43.7	26	25	1
	pZM1252603	NC_CP003713.1	37.06	42.4	51	50	1
	pZM1252604	NC_CP003714.1	32.4	43.7	27	24	3
	pZM1252605	NC_CP003715.1	32.8	43.3	23	20	3
NRRL B-14023	pZM1402301	NC_CP003716.1	33.92	42.3	29	28	1
	pZM1402302	NC_CP003717.1	30.95	43.7	27	26	1
	pZM1402303	NC_CP003718.1	37.06	42.4	51	49	2
	pZM1402304	NC_CP003719.1	32.8	43.3	23	20	3
NCIMB 11163	pZA1001	NZ_CP003712.1	53.38	42.3	54	52	2
	pZA1002	NZ_CP003713.1	40.82	43.8	32	32	-
	pZA1003	NZ_CP003714.1	4.55	36.4	6	5	1
ATCC 10988	pZMOB01	NZ_CP003716.1	32.48	43.5	30	27	3
	pZMOB02	NZ_CP003717.1	32.28	45.4	29	25	4
	pZMOB03	NZ_CP003718.1	31.69	43.2	25	24	1
	pZMOB04	NZ_CP003719.1	18.46	41.8	27	26	1
	pZMOB05	NC_013784.1	4.02	37.6	3	3	-
	pZMOBP6	NC_013785.1	2.75	41.3	2	2	-
ATCC 29191	pZZ6.01	NC_013786.1	18.35	41.0	23	20	3
	pZZ6.02	NC_013787.1	14.95	42.2	18	18	-
	pZZ6.03	NC_013788.1	13.74	44.2	11	10	1
ATCC 29192	pZYMOP01	NC_015715.1	37.39	41.0	37	38	1
	pZYMOP02	NC_015716.1	34.16	44.0	33	34	1
NRRL B-1960	pZMO1960-1	CP021791	34.46	418	37	38	1
	pZMO1960-1A	CP021792	1.73	38.2	33	34	1

### Comparison at the genomic level

Comparative genome analysis was performed on 8 *Z*. *mobilis* genomes to provide a picture of the genetic diversity within this species. All strains analyzed in this study, were shown in Table 1, which includes genome size, GC content, and number of plasmids.

**Fig 1:**
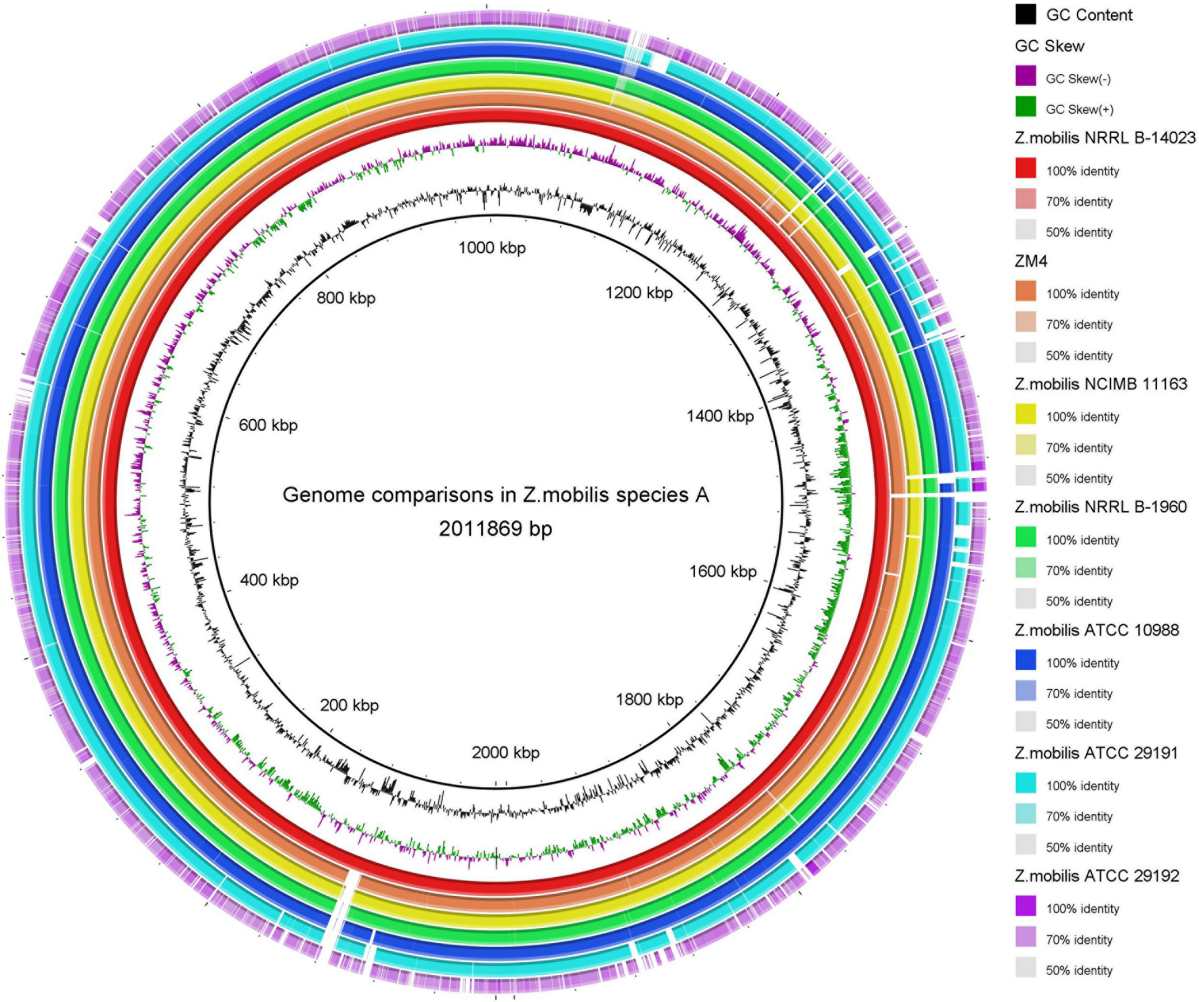
Whole genome comparison in 8 *Z*. *mobilis*. Whole-genome comparison of 8 strains (alignment reference genome: NRRL B-12526). From outer to inner ring: NRRL B-12526, NRRL B-14023, ZM4, NCIMB 11163, NRRL B-1960, ATCC 10988, ATCC 29191, ATCC 29192.The color intensity in each ring represents the BLAST match identity.

A circular genome map for each genome was constructed by using the BLAST Ring Image Generator [34]. A visual inspection the circular alignment of the genomes of *Z*. *mobilis* (Fig 1) revealed a relatively high sequence similarity; especially the region of 100–1000 kbp. It was identical in 6 isolated strains to the alignment reference genome of NRRL B-12526, except for *Z*. *mobilis* ATCC 29192. In these regions we found genes related to the Entner-Doudoroff (ED) pathway, the carbohydrate metabolic process, the nitrogen compound metabolic process and the biosynthetic process. These include amino acid biosynthesis, NAD biosynthesis, carbohydrate biosynthesis, fatty acid biosynthesis and coenzyme A biosynthesis.

The regions at 1000–2000 kbp demonstrated more than 96% identity with other Z. *mobilis* strains to the alignment reference genome of NRRL B-12526, with some variable chromosome. We observed a regions at 90-100kbp in the chromosome of NRRL B-12526 that were not apparently present in the genomes of other strains, except for NRRL B-14023(Fig 1). The only genes in the region whose function could be predicted were lipoprotein transporter, deoxyribonucleoside diphosphate metabolic process, ATPase activity. The region of 1320-1330kbp and 1480-1520kbp were absent in other strains, too. The proteins differentially present in the 1320-1330kbp regions were involved in Arylesterase, cyanate transporter, CysJI operon and Shikimate dehydrogenase. While the proteins in regions 1480-1520kbp were glutamine amidotransferase and HTH-type transcriptional regulatory protein. In our lab, comparison of ethanol production was made among 4 *Z*. *mobilis* (ZM4, ATCC 29191, NRRL B-14023 and NRRL B-12526) in sweet potato media with different glucose concentrations. The results showed that *Z*. *mobilis* NRRL B-12526 could rapidly metabolize substrates of glucose into ethanol (unpublished data). The differences between strains in physiological and fermentation ability may be related to its distinctive gene expression. However, the presences or absences of these distinctive genes were not sufficient to explain their different ethanol fermentation efficiencies, and therefore further experiments are necessary to fully elucidate it.

Interestingly, we found a large gap in *Z*. *mobilis* ATCC 29291 between 1120–1140 kbp (Fig 1). In these regions, the proteins were absent in Z. mobilis ATCC 29291 compared to other strains. These proteins included putative endoribonuclease L-PSP, polar-differentiation response regulator divK, Unfoldase HslU, Hup, ATP synthase subunit alpha and beta and F-type ATPase subunit delta and gamma. Thus we inferred that because these genes were absent, the ATCC 29191 genome was the smallest of the 8 strains (Table 1). Because these genes were absent, *Z*. *mobilis* ATCC 29291 was superior to other *Z*. *mobilis* strains in levan (polyfructan) production [15].

We found ZM4 had regions, such as 1980-2030kbp with higher GC contents that were not present in the whole genome sequences of other strains (S1 Fig). Chaperonin Cpn10 and GroEL, transcriptional regulator XRE family and GntR family were found in these regions, according to their location in the genome of the alignment reference genome ZM4.

There were some differences between the genome of ATCC29192 and the other stains. We found that more than 40 regions in genome sequence of strain ATCC 29192 were absent when compared to the other strains were absent (Fig 1). The more evident gaps(missing regions) were visible at positions 570–610, 680–710, 1190–1220, 1240–1280, 1380–1400, 1460–1490 and 1760–1770kbp (Fig 1). In these absent regions, ATCC29192 lacked transcription factors of the MarR family and the TetR family and a series of nitrogen fixation-associated proteins. The regions at positions 640–650, 1140–1150, 1170–1230, 1240–1280, 1460–1490 and 1760–1770kbp (S1 Fig) were uniquely present in ATCC29192. Among these *Z*. *mobilis* strains, only ATCC29192 was able to encode ferritin δ chain and a specific nitrogen reductase. It is possible that the distinctive genes of these regions caused ATCC 29192 to exhibit distinct traits, such as low oxygen tolerance and increase nutritional requirements [20].

**Fig 2:**
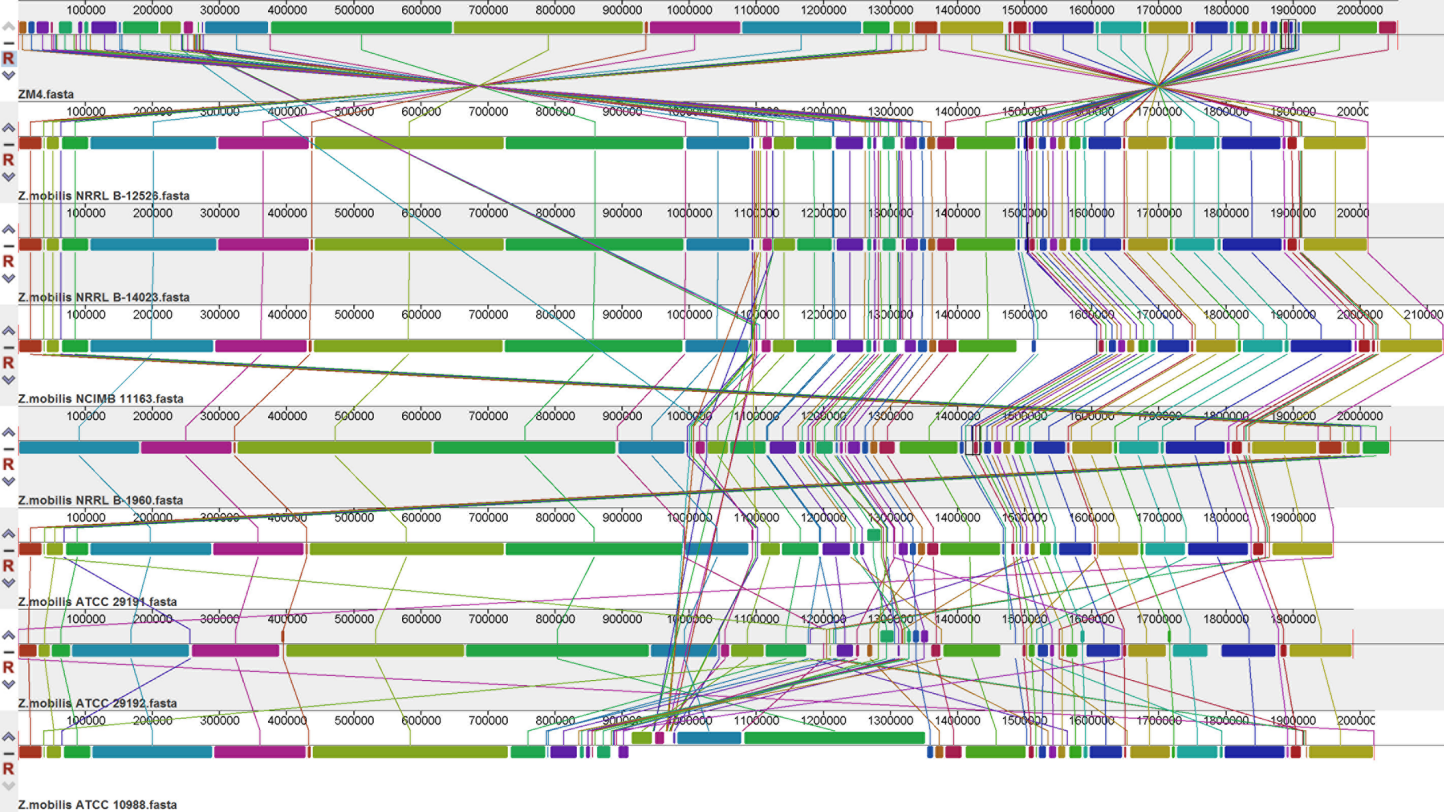
Complete genome alignment of 8 *Z*. *mobilis*. Colored outlined blocks surround regions of the genome sequence that aligned with part of another genome. Homologous regions that are conserved are shown in the same colors. The coloured bars inside the blocks are related to the level of sequence similarities. Lines link blocks with homology among genomes.

To detect chromosomal rearrangements, deletions, and duplications among strains of *Z*. *mobili*s, the alignment of the genomes of all strains were analyze using MAUVE (Fig 2). As showing in Fig 2,there was considerable conservation of the 8 genomes was revealed, although some serotype-specific regions were observed (Fig 2). However, the position of conserved regions in the ZM4 genome was rotated 180 degrees compared to other strains (Fig 2). Perhaps, the genome of ZM4 maybe had rearrangements occur during evolution. In addition, since the genome sequences were obtained by next-generation” sequencing, which was performed on the Illumina HiSeq 2000 platform. Thus the difference between the genome was likely to be related to the assembly method. However, the ZM4 genome used for the reference genome was still appropriate and convincing [12, 13]. Two subtype-specific insertions were observed: the type 1-specific 1500-1600kbp (NCIMB 11163) insertion and type 2-specific 1300–1400 kbp (ATCC 29192 and ATCC 29191) insertion and inversions (Fig 2). The region of 800–1000 kbp in ATCC 29191 was shifted compared to the other strains. While there were a few structural rearrangements and shifts of the corresponding chromosomes in some strains, the changes did not appear to affect protein-coding genes.

### Comparison of *Z*. *mobilis*-harboring plasmids

Plasmids are an important ways to obtain foreign genetic components. Analysis of the genome sequences of *Z*. *mobilis* strains showed that there were significant differences in the types and numbers of plasmids in the types and numbers, as well as the size and number of plasmids between the different *Z*. *mobilis* strains (Table 2). A comparison of plasmids genes was carried out in 8 strains of *Z*. *mobilis* by the program MAUVE. Homologous regions are indicated by the same colors (S2 Fig). Although the sizes and numbers of plasmids were different between various *Z*. *mobilis* strains, the plasmid genes of plasmids were highly conserved, especially the plasmids in *Z*. *mobilis* ZM4, NRRL B-12526, NR RL B-14023, NCIMB 11163 and NRRL B-1960.For example, the *Z*. *mobilis* NRRL B-1960 plasmid sequence of pZMO1960-1A is identical to the *Z*. *mobilis* NCIMB 11163 plasmid pZMO1A, which the complete genome sequences were most closely related between these strains[16].The sequences of plasmids pZZM401, pZM1252603 pZM1402303,pAZ1001,pZMOB04 and pZMOB05 were relatively similar. However, the plasmid sequences of *Z*. *mobilis* ATCC 29192 were different than the other strains. For example, plasmid pZYMOP01 of ATCC 29192 carries a CRISPR repeat region. From these results, we found a high level of homology between the complete genome of all sequences, and we found the plasmids sequences of *Z*. *mobilis*-harboring plasmids were also conserve.

### Phylogenetic comparisons of whole genomes and plasmids among *Z*. *mobilis* strains

**Fig 3:**
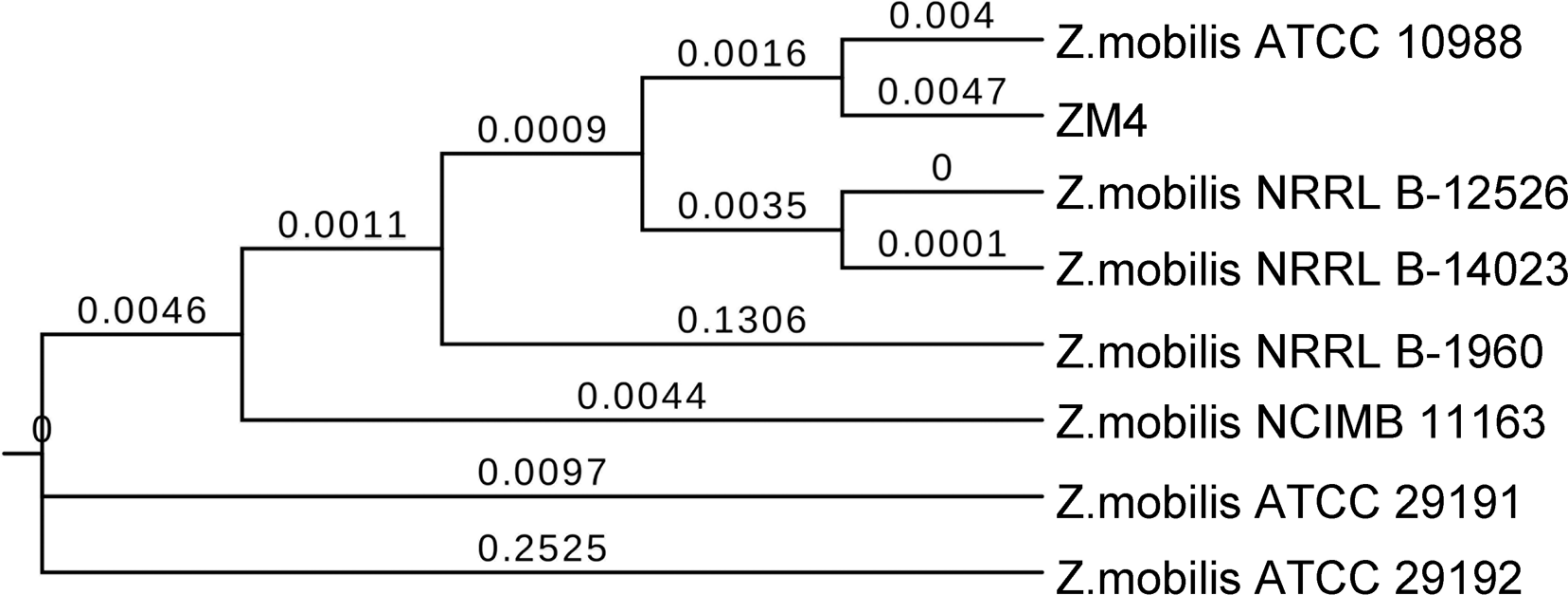
Phylogenetic tree showing the relationship among 8 *Z*. *mobilis* strains based on complete genome sequences. Branch length values were shown for branches separating different strains.

**Fig 4:**
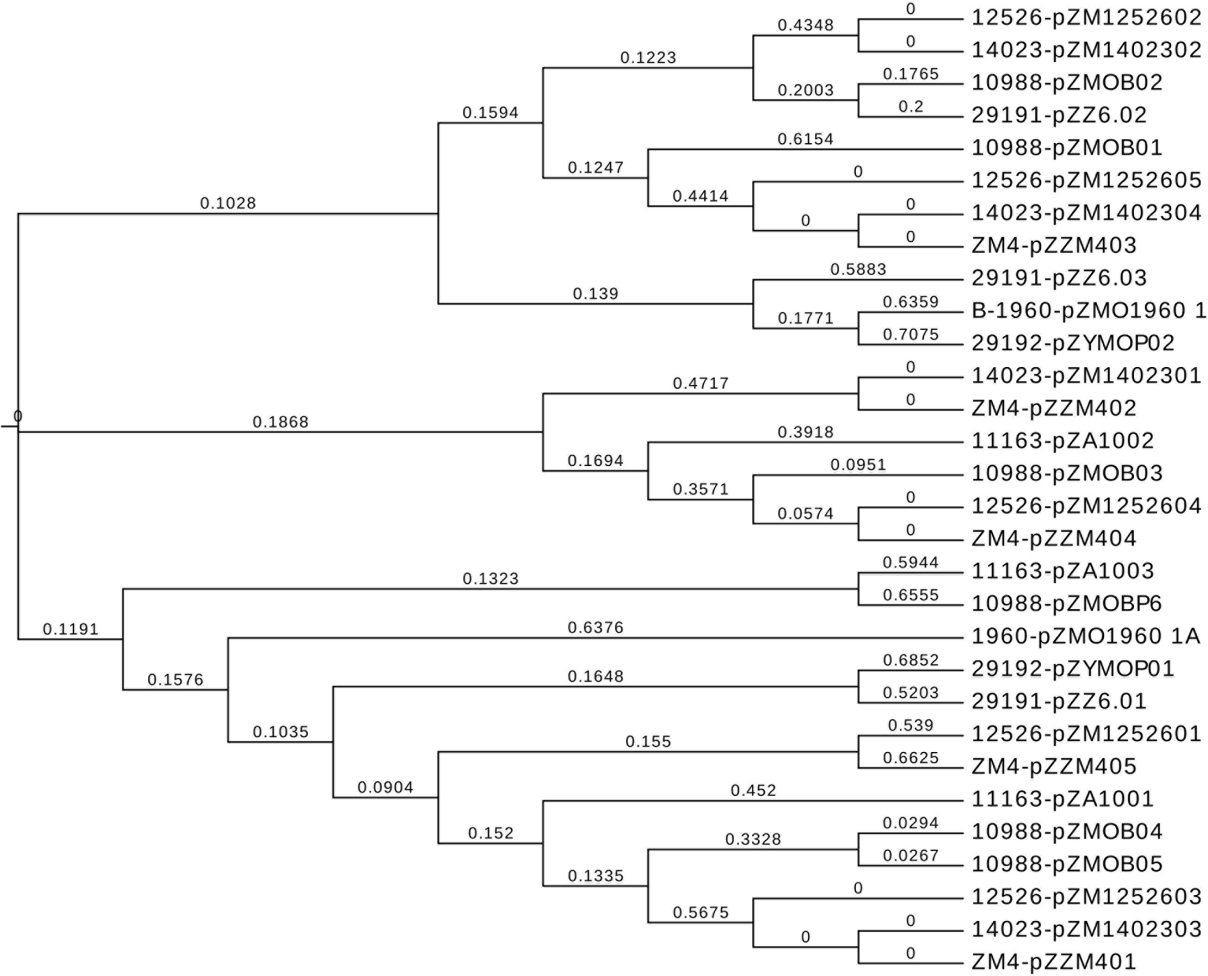
Phylogenetic tree of plasmids sequences of 8 *Z*. *mobilis* strains. Branch length values were shown for branches separating different plasmids.

A phylogenetic tree of 8 the sequenced *Z*. *mobilis* strains was constructed based on the complete genome sequences using MMEA. The phylogenetic tree analysis indicated that NRRL B-12526 and NRRL B-14023, ZM4 and ATCC10988 gathered in a cluster, which suggests they could share a similar evolutionary path. ATCC29191 and ATCC29192 had a distant genetic relationship with the other strains. Particularly, the branch length (value 0.2525) of ATCC29192 was bigger than the others strains, which indicates that it is phylogenetically more distant (Fig 3). The phylogenetic relationships between the plasmids of all *Z*. *mobilis* strains were constructed (Fig 4). As expected, plasmids from the same cluster of strains showed close evolutionary relationships, such as plasmids pZZM403, pZM1402304, and pZM1252605.Although the strains ATCC29192 had a relationship distant from the other strains, the plasmid pZYMOP02 was clustered with plasmid pZZM402, pZM1402301 and pZM1252601.Additionally, pZYMOP01 was genetically closer to plasmid pZZM401, pZM1402303, and pZM1252603(Fig 4).

### Clustered regularly interspaced short palindromic repeats (CRISPRs)

**Fig 5:**
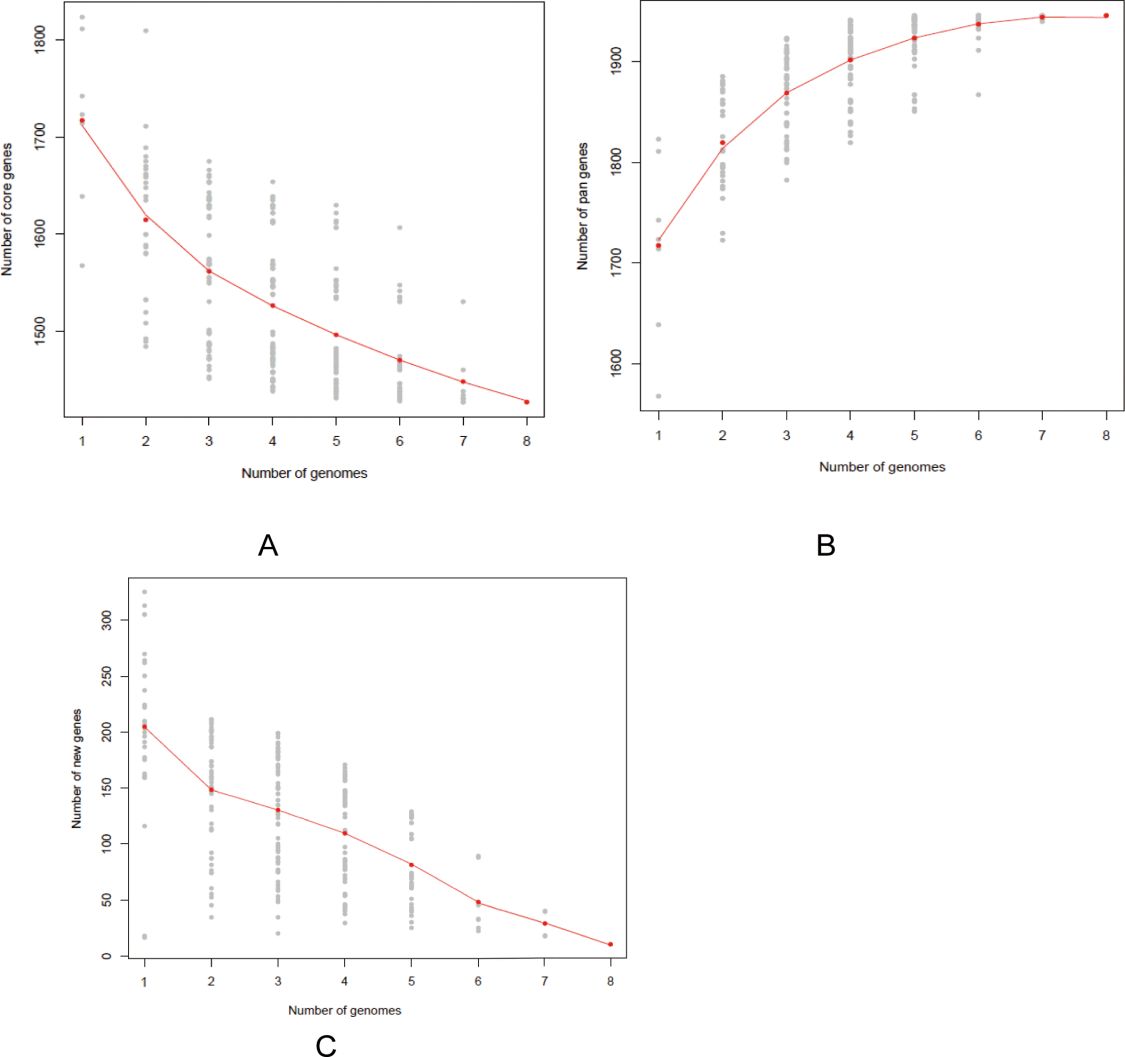
Pan-genome calculations of the conserved core, predicted new genes of 8 *Z*. *mobilis* strains. (A) Z. *mobilis* core genome. Each point represents the number of conserved genes between genomes. The red line demonstrated the exponential decay model based on the median value for conserved genes when increasing numbers of genomes were compared. (B) *Z*. *mobilis* pan-genome. The red line showed the exponential model based on the mean value of pan genes (C) Number of *Z*. *mobilis* new predicted gene clusters. The red line showed the exponential decay model based on the median value for new genes when increasing numbers of genomes were compared.

Eight *Z*. *mobilis* strains had 3–5 types of CRIPSR repeats, CRISPR1, CRISPR2 and CRISPR3 were ubiquitous, but CRISPR4 and CRISPR5 were only present in the strains ATCC10988 and ATCC29191, respectively (Fig 5). The probability and types of phage infection may be different because of changing the living environment. Therefore, when the numbers and types of phage infection are increased, the number of CRISPR sites of in the *Z*. *mobilis* genome will be increased [41–43].

In the CRISPR-Cas systems of *Z*. *mobilis* strains, there were 13 different spacer profiles and 5 types repeats were identified. NRRL B-12526 and NRRL B-14023 shared the three same CRISPR repeat and spacers numbers (spacers 7, 4, and 5) (S1 Table), indicating that the strains invaded by the same phage or plasmid [43]. Interestingly, the CRISPR repeats (TTTCTAAGCTGCCTGTGCGGCAGTGAAC and GTTCACTGCCGCACAGGCAGCTTAGAAA) were common to all 8 *Z*. *mobilis*. However, the CRISPR repeats (CCAGAAATACTGCACTCGCTGTAATAGCCCCGATCTCTCAC) was only found in *Z*. *mobilis* ATCC10988; and the CRISPR repeat (CGGTTCATCCCCGCGTGGGCGGGGAACAC) was only present in *Z*. *mobilis* ATCC29192.

Overall, there were a lot of common features among the CRISPR/Cas systems of *Z*. *mobilis* strains, which were conserved. However, the strains of ATCC29192, ATCC29191 and ATCC10988 acquired some new characteristics during evolutionary.

### Core and pan-genome analysis of *Z*. *mobili*

**Fig 6:**
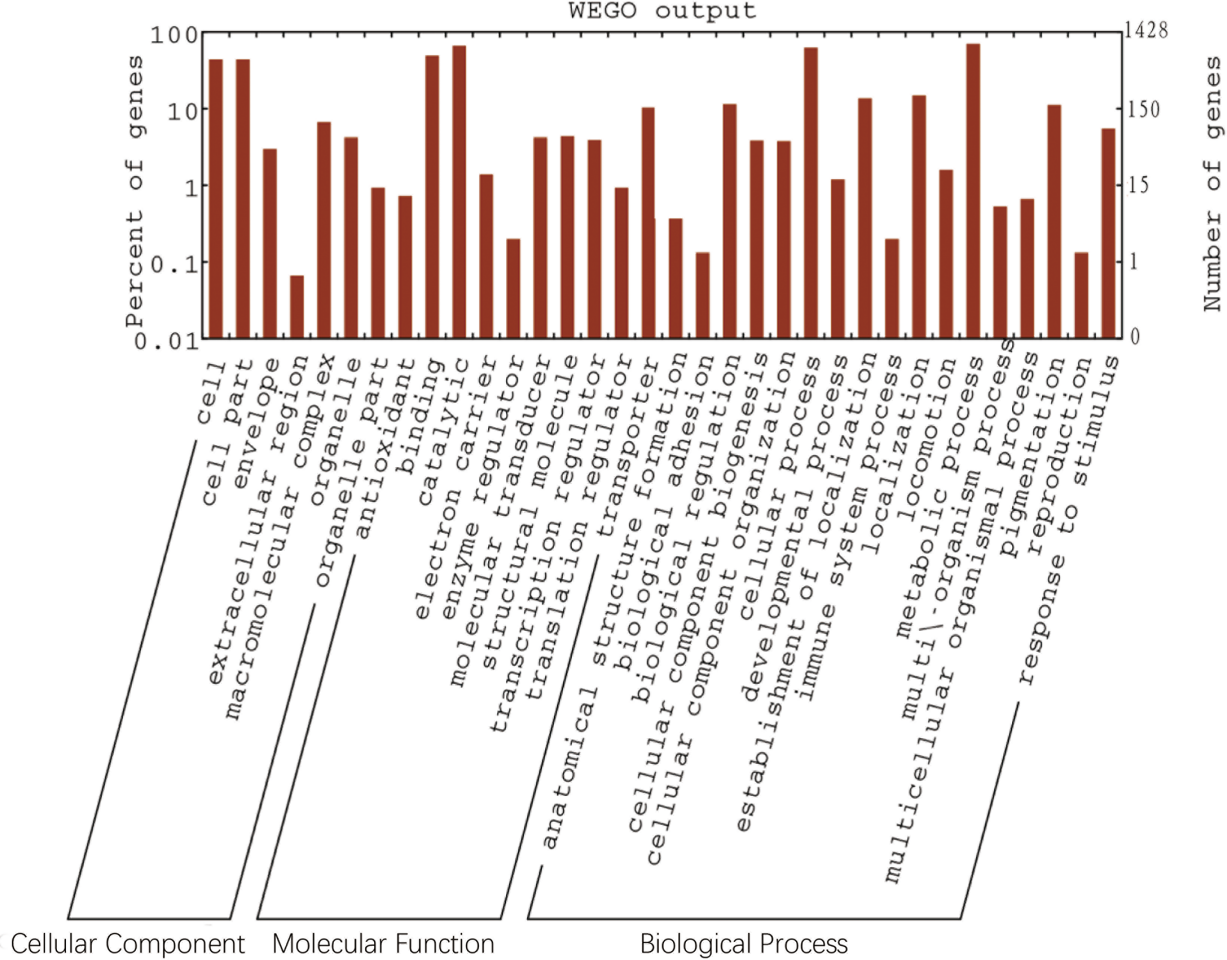
Comparison of COG functional categories of core genes. The left Y-axes meant percent of gene annotated in each GO term (gene number in each GO term divided by gene number in all GO terms). The right y-axes meant gene number annotated in each GO term. The left y-axes meant percent of gene annotated in each GO term (gene number in each GO term divided by gene number in all GO terms). The right y-axes meant gene number annotated in each GO term.

Pan-genome refers to the sum of genes that can be found in a given bacterial species. This included the core genome (genes shared by all strains) and the dispensable genome (genes absent from one or more strains, and genes that are unique to each strain) [44–46]. The core genome encodes essential functions related to the basic biology of the species and genes shared by all strains [47]. The dispensable genome contributes to species’ diversity and the genes that are absent from one or more strains, and the genes that are unique to each strain [47]. To understand the basic biology and population genetics of *Z*. *mobilis*, the core and dispensable genomes were analyzed by OrthoMCL version 2.0. The size of the pan-genome was 1945 genes shared by 8 strains. The core genome included 1428 genes, which account for 73.41% of CDSs of these strains. There were 517 accessory genes, including 507 dispensable genes and 10 unique genes. These accounted for 26.59% of the total CDSs from all 8 genomes. However, the distribution the core gene number in each strain varied considerably (Fig 5A). These core genes are related to carbohydrate metabolism, replication, transcription and translation. The extrapolated curve plateaus at a value of about 1900 with 8 genome sequences, and it can be inferred that the *Z*. *mobilis* had a closed pan-genome (Fig 5B). The genome structure of the strain of *Z*. *mobilis* was very conservative. There were 10 new genes found in different strains of *Z*. *mobilis*, 6 new genes in NCIMB 11163, 3 new genes in ATCC 29191 and 1 new gene in ATCC 29192 (Fig 5C). We found that these 1428 core genes were also subjected to COG functional classification (Fig 6). These 1428 core gene were more often associated with the metabolic process, catalytic binding, cellular process, transporters and biological regulation (Fig 6).

## Conclusions

In summary, the comparative genomic analyses indicated that these strains shared a conserved core chromosomal backbone, and they shared quite high homology with an average of 96% sequence identity. ZM4, NRRL B-12526, NRRL B-14023, NRRL B-1960 and NCIMB 11163 shared an extremely short evolutionary relationship in the phylogenetic tree. Furthermore, strains ATCC10988, ATCC29191 and ATCC29192 had a far distant relationship. The size of the *Z*. *mobilis* pan-genome was 1945 genes, and it includes 1428 core genes and 517 accessory genes, which had a closed pan-genome. In the current study, we established a link between the sequenced complete genome sequences of *Z*. *mobilis*. We also found similarities and differences between the genomes of these strains, which indicated that *Z*. *mobilis* strains were conserved during evolutionary.

## Supporting information

S1 Fig Whole genome comparison in 8 *Z*. *mobilis* strains. (A)Whole-genome comparison of 8 strains (alignment reference genome: ZM4). From outer to inner ring: ZM4,NRRL B-12526,NRRL B-14023, NCIMB 11163, NRRL B-1960,ATCC 10988, ATCC 29191,ATCC 29192. (B)Whole-genome comparison of 8 strains (alignment reference genome: ATCC 29192). From outer to inner ring: ATCC 29192,NRRL B-12526,NRRL B-14023,ZM4, NCIMB 11163, NRRL B-1960,ATCC 10988, ATCC 29191.The color intensit yin each ring represents the BLAST match identity.
(TIF)

S2 Fig Plasmid alignments of 8 *Z*. *mobilis* strains. Colored outlined blocks surround regions of the plasmid sequences that aligned with part of another genome. The coloured bars inside the blocks are related to the level of sequence similarities.
(TIF)

S1 Table CRISPRs found in 8 *Z*. *mobilis* strains. (DOCX)
