# Supplementary material for: Genome comparison of different Zymomonas mobilis strains provides insights on conservation of the evolution
Source: PLoS One. 2018 Apr 25;13(4):e0195994. doi: 10.1371/journal.pone.0195994 (PMC5919020; doi:10.1371/journal.pone.0195994)
Supplement: S1 Table — (DOCX) [file pone.0195994.s003.docx]

S1 Table

| Z.mobilis Strain | CRISPR Number | CRISPR kinds | CRISPR Position | CRISPR Length(bp) | Spacer Number | Special Spacer* (DR consensus) | *cas* genes | Type |
| --- | --- | --- | --- | --- | --- | --- | --- | --- |
| ZM4 = ATCC 31821 | 3 | CRISPR1 | 242955 -1243466 | 511 | 8 | GTTCACTGCCGCACAGGCAGCTTAGAAA | *cas*1, *cas*3,*csy*1, *csy*2,  *csy*3, *csy*4 | I-F |
|  |  | CRISPR2 | 1590356-1590747 | 391 | 6 | GTTCACTGCCGCACAGGCAGCTTAGAAA |  |  |
|  |  | CRISPR3* | 1590917-1591005 | 88 | 1 | GTTCACTGCCGCACAGGCAGCTTAGAAA |  |  |
| [CP4 = NRRL B-14023](http://www.ncbi.nlm.nih.gov/genome/898?genome_assembly_id=169871) | 3 | CRISPR1 | 131560 - 132020 | 460 | 7 | TTTCTAAGCTGCCTGTGCGGCAGTGAAC | *cas*1, *cas*3,  *csy*1, *csy*2,  *csy*3, *csy*4 | I-F |
|  |  | CRISPR2 | 1255177 -1255445 | 268 | 4 | GTTCACTGCCGCACAGGCAGCTTAGAAA |  |  |
|  |  | CRISPR3 | 1808132 -1808460 | 328 | 5 | TTTCTAAGCTGCCTGTGCGGCAGTGAAC |  |  |
| NRRL B-12526 | 3 | CRISPR1 | 131560 -132020 | 460 | 7 | TTTCTAAGCTGCCTGTGCGGCAGTGAAC | *cas*1, *cas*3,  *csy*1, *csy*2,  *csy*3, *csy*4 | I-F |
|  |  | CRISPR2 | 1254478-1254746 | 268 | 4 | GTTCACTGCCGCACAGGCAGCTTAGAAA |  |  |
|  |  | CRISPR3 | 1807530 -1807858 | 328 | 5 | TTTCTAAGCTGCCTGTGCGGCAGTGAAC |  |  |
| NCIMB 11163 | 3 | CRISPR1 | 126368 -127178 | 810 | 13 | TTTCTAAGCTGCCTGTGCGGCAGTGAAC | *cas*1, *cas*3,  *csy*1 *csy*2,  *csy*3, *csy*4 | I-F |
|  |  | CRISPR2 | 1255057 -1256050 | 993 | 16 | GTTCACTGCCGCACAGGCAGCTTAGAAA |  |  |
|  |  | CRISPR3 | 1909883-1911666 | 1783 | 29 | TTTCTAAGCTGCCTGTGCGGCAGTGAAC |  |  |
| pZA1002 (Plasmid of NCIMB 11163) | 1 | CRISPR1 | 33670 --36950 | 3280 | 49 | GTTTCAATCCACGCCTCCGCGAAGGAGGCGAC | *cas*1, *cas*2,*cas*3, *cas*4,  *csd*1, *csd*2 | I-C |
| ATCC 10988 | 4 | CRISPR1 | 125014 -125404 | 390 | 6 | TTTCTAAGCTGCCTGTGCGGCAGTGAAC | *cas*1, *cas*3  *csy*1, *csy*2,  *csy*3 | I-F |
|  |  | CRISPR2* | 579550 -579677 | 127 | 1 | CCAGAAATACTGCACTCGCTGTAATAGCCCCGATCTCTCAC |  |  |
|  |  | CRISPR3 | 830110 - 830314 | 204 | 3 | ACTGCCGCACAGGCAGCTTAGAAA |  |  |
|  |  | CRISPR4 | 1811916 -1812426 | 510 | 8 | TTTCTAAGCTGCCTGTGCGGCAGTGAAC |  |  |
| ATCC 29191 | 5 | CRISPR1 | 125613 -125820 | 207 | 3 | GTTCACTGCCGCACAGGCAGCTTAGAAAA | *c cas*1, *cas*3,  *csy*1, *csy*2,  *csy*3, *csy*4 | I-F |
|  |  | CRISPR2 | 1235875-1236083 | 208 | 3 | TTTCTAAGCTGCCTGTGCGGCAGTGAAC |  |  |
|  |  | CRISPR3 | 1756727-1757238 | 511 | 8 | TTTCTAAGCTGCCTGTGCGGCAGTGAAC |  |  |
|  |  | CRISPR4* | 1793736-1793801 | 65 | 1 | GTTCACTGCCGCACAGGCAGCTTAGAAA |  |  |
|  |  | CRISPR5* | 1794006-1794184 | 178 | 2 | GTTCACTGCCGCACAGGCAGCTTAGAAA |  |  |
| ATCC 29192 | 3 | CRISPR1 | 1188329-1189028 | 699 | 11 | CGGTTCATCCCCGCGTGGGCGGGGAACAC | *cas*1, *cas*2,  *cas*5, *cse*1,  *cse*2, *cse*3, *cse*4 | I-E |
|  |  | CRISPR2 | 1310992-1312057 | 1065 | 17 | CGGTTCATCCCCGCGTGGGCGGGGAACAC |  |  |
|  |  | CRISPR3 | 1183182-1183269 | 87 | 1 | GTTCATCCCCGCGTGGGCGGGGAACAC |  |  |
| pZYMOP01 (Plasmid of ATCC 29192) | 1 | CRISPR1 | 11752 --12839 | 1087 | 16 | GTTTCAATCCACGCCTCCGCGAAGGAGGCGA | *cas*1, *cas*2,*cas*3, *ccas*4,*csd*1, *csd*2 | I-C |
| NRRL B-1960 | 3 | CRISPR1 | 14970-16385 | 1415 | 23 | TTTCTAAGCTGCCTGTGCGGCAGTGAAC | *cas*1 | I-F |
|  |  | CRISPR2 | 1154817-1156350 | 1533 | 25 | TTTCTAAGCTGCCTGTGCGGCAGTGAAC | *csy*1 *csy*2 |  |
|  |  | CRISPR3 | 1723946-1725188 | 1242 | 20 | TTTCTAAGCTGCCTGTGCGGCAGTGAAC | *csy*3 |  |

[*****](http://crispr.u-psud.fr/cgi-bin/crispr/advRunCRISPRFinder.cgi#footnote)**: Questionable CRISPRs**
